# Putative biomarkers for predicting tumor sample purity based on gene expression data

**DOI:** 10.1186/s12864-019-6412-8

**Published:** 2019-12-27

**Authors:** Yuanyuan Li, David M. Umbach, Adrienna Bingham, Qi-Jing Li, Yuan Zhuang, Leping Li

**Affiliations:** 1 Biostatistics and Computational Biology Branch, National Institute of Environmental Health Sciences, Research Triangle Park, North Carolina 27709, USA MD A3-03, Durham, NC 27709 USA; 2 Department of Immunology, Duke University, Durham, North, Carolina 27710 USA

**Keywords:** Tumor purity, RNA-seq, Gene expression, XGBoost, Gradient boosted trees, And machine learning

## Abstract

**Background:**

Tumor purity is the percent of cancer cells present in a sample of tumor tissue. The non-cancerous cells (immune cells, fibroblasts, etc.) have an important role in tumor biology. The ability to determine tumor purity is important to understand the roles of cancerous and non-cancerous cells in a tumor.

**Methods:**

We applied a supervised machine learning method, XGBoost, to data from 33 TCGA tumor types to predict tumor purity using RNA-seq gene expression data.

**Results:**

Across the 33 tumor types, the median correlation between observed and predicted tumor-purity ranged from 0.75 to 0.87 with small root mean square errors, suggesting that tumor purity can be accurately predicted υσινγ expression data. We further confirmed that expression levels of a ten-gene set (*CSF2RB*, *RHOH*, *C1S*, *CCDC69*, *CCL22*, *CYTIP*, *POU2AF1*, *FGR*, *CCL21*, and *IL7R*) were predictive of tumor purity regardless of tumor type. We tested whether our set of ten genes could accurately predict tumor purity of a TCGA-independent data set. We showed that expression levels from our set of ten genes were highly correlated (ρ = 0.88) with the actual observed tumor purity.

**Conclusions:**

Our analyses suggested that the ten-gene set may serve as a biomarker for tumor purity prediction using gene expression data.

## Background

The tumor microenvironment consists of non-cancerous stromal cells present in and around a tumor; these include immune cells, fibroblasts, and cells that comprise supporting blood vessels and others. Tumor microenvironment plays an important role in tumor initiation, progression, and metastasis (for recent reviews, see [1, 2]).

Most genomic and genetic studies of cancer are carried out on tumor tissue samples that are heterogenous in nature. The Cancer Genome Atlas (TCGA) provided comprehensive datasets for more than 10,000 samples in more than 30 tumor types [3]. Those studies provide valuable information about genomic changes in tumor samples compared to normal samples. However, teasing out cell-type-specific information from those heterogeneous samples remains a challenge.

Knowing the cell-type composition of a tumor and how those cell types interact with each other in the tumor microenvironment is pivotal for understanding tumor initiation, progression, and metastasis. While understanding the microenvironment is challenging, methods for addressing cell composition in tumor samples, such as single cell technologies, are starting to emerge. For instance, Zheng et al. profiled the infiltrating T cells in liver cancer [4], Puram et al. [5] surveyed the tumor ecosystems in head and neck cancer, and Kararaayvaz et al. analyzed leukocyte composition in triple negative breast cancer [6]. All of these studies used single cell RNA-seq sequencing (scRNA-seq) techniques. It is conceivable that single cell sequencing technologies will be widely applied to dissect the tumor microenvironment.

Computational methods directed at deconvolving cell-type-specific signals in heterogeneous tissue samples have also been developed (for a recent review, see [7]). Currently, several computational methods can estimate the proportion of tumor cells in a tumor sample (often referred to as “tumor purity”). Perhaps, the most well-known algorithm is ABSOLUTE [8], which uses copy number variation in tumor samples compared to normal samples to infer tumor purity and ploidy. ABSOLUTE, which is often considered as the “gold standard” for performance comparison, provided tumor purity values for many samples from the 33 TCGA tumor types [9]. Besides copy number variation, methods that use DNA methylation data [10–13] or expression data for a set of pre-selected stromal genes [14] have also been developed to infer tumor purity. Purity estimates by those methods appear to have a reasonable concordance [15].

Similarly, tumor purity estimates have been used to assess the abundance of tumor-infiltrating immune cells in tumor samples. For example, Li et al. [16] developed a computational method to estimate the abundance of six tumor-infiltrating immune cell types (B cells, CD4 T cells, CD8 T cells, neutrophils, macrophages, and dendritic cells) in tumor samples. Iglesia et al. [17] assessed immune cell infiltration in 11 TCGA tumor types using a set of immune signature genes from [18]. Senbabaoglu et al. [19] employed 24 immune cell-type-specific gene signatures from [18] to computationally infer the immune cell infiltration levels in tumor samples and defined a T-cell-infiltration score, an overall immune-infiltration score, and an antigen-presenting-machinery score to highlight the immune response differences between kidney cancer and 18 other tumor types from TCGA.

For high-dimensional data where the number of features is much greater than the number of samples (*p* > > *n*), there may not exist a single set of features that can deliver the optimal/suboptimal performance. For those data, repeated cross-validations may be needed and aggregated prediction from an ensemble of ensembles (boosting) is usually preferred [20]. Ensemble learning generates multiple prediction models from the training data, each with a different feature subset. By using multiple learners, the generalization ability of an ensemble can be much better than any of the individual constituent learning algorithms [21–23]. Popular ensemble learning algorithms include bagging [24], boosting [25, 26], and stacking [27]. Bagging trains a number of learners each from a different bootstrap sample and combines the predictions using a majority vote. Random Forest [28] is a popular technique in this category. Boosting iteratively adds new weak learners to correct the mistakes made by previous learners and collectively, the weak learners become a strong learner. The most common implementation of boosting is Adaboost [29] and Gradient Boosting Machines (GBM) [30]. In stacking, one generates multiple different types of models to build intermediate predictions, which are subsequently combined by a second-level meta-learner. Generally speaking, ensemble learning consistently outperforms non-ensemble-based methods.

XGBoost (eXtreme Gradient Boosting) is an ensemble learning algorithm [31]. XGBoost extends simple CARTs (Classification And Regression Trees) by incorporating a statistical technique called boosting. Instead of building one tree, boosting improves prediction accuracy by building many trees and then aggregating them to form a single consensus prediction model [32]. XGBoost creates trees by sequentially using the residuals from the previous tree as the input for the subsequent tree. In this manner, subsequent trees improve overall prediction by modeling the errors of the previous tree. When the loss function is least squares, this sequential model building process can be expressed as a form of gradient descent that optimizes prediction by adding a new tree at each stage to best reduce the loss [33]. The addition of new trees is stopped either when a pre-specified maximum number of trees is reached or when the training errors do not improve for a pre-specified number of sequential trees. Both the approximation accuracy and execution speed of gradient boosting can be substantially improved by incorporating random sampling; this extended procedure is called “stochastic gradient boosting” [30]. Specifically, for each tree in sequence, a random subsample of the training data is drawn without replacement from the full training data set. This randomly selected subsample is then used in place of the full sample to fit the tree and compute the model update. XGBoost is an optimized distributed gradient boosting that achieves state-of-the-art prediction performances [31]. XGBoost uses second order approximation of the loss functions for faster convergence compare to traditional GBMs. XGBoost has been successfully used in mining gene expression data [34].

Previously, we used XGBoost for pan-cancer classification based on gene expression data [35]. In this work, we used XGBoost to select a subset of genes whose gene expression levels can predict tumor purity. Our work was prompted by the observation that expression of many immune genes was negatively correlated with tumor purity where tumors with high immune gene expression tended to have fewer cancer cells and vice versa. We applied XGBoost to 33 TCGA tumor types for which both RNA-seq gene expression and ABSOLUTE tumor purity estimates [8] were available. We carried out several analyses for all tumor types combined (pan-cancer) using all genes. We showed that XGBoost can accurately predict tumor purity values using gene expression data alone. By considering how useful or important each gene was to the model’s prediction, we selected the top 10 most important genes as putative markers for tumor purity prediction. For TCGA data and an independent set of non-TCGA samples, we showed that predictions based on the expression levels of only these top ten genes is almost as accurate as predictions based on using all genes. We propose that these ten genes may serve as biomarkers for tumor purity prediction.

### Data

We downloaded the processed TCGA RNA-seq gene expression data (RNA final) from the Pan-Cancer Atlas Publication website (https://gdc.cancer.gov/about-data/publications/pancanatlas) for 11,069 samples from 33 tumor types. The data were pre-processed and normalized by TCGA to remove all batch effects [9]. We log_2_-transformed the normalized read counts (per million reads mapped) for RNA-seq data (all values less than 1 were assigned value 1 before transformation). We filtered out genes with missing values or zero variances. The number of remaining genes was 17,170. We also removed 8 duplicate samples that were from the same patients.

The tumor purity estimates by ABSOLUTE [8] for tumor tissue samples from 33 TCGA tumor types were obtained from [9]; these purity estimates are summarized for each tumor type in Additional file 1: Figure S1. The observed purity estimates are bound between 0 and 1. However, the predicted value can be greater than 1 or smaller than 0. To prevent the predicted value from being outside of these boundaries, we applied the logit transformation to map the original purity values in the range of [0, 1] to the real line, thereby enhancing concordance with the regression for continuous outcomes implemented in XGBoost. We re-assigned the purity values of 1.00 to 0.9975, which is the value midway between the two biggest purity values. To express logit-transformed predicted purity values derived from XGBoost back in the original scale, we applied the inverse logit transformation.

**Fig. 1:**
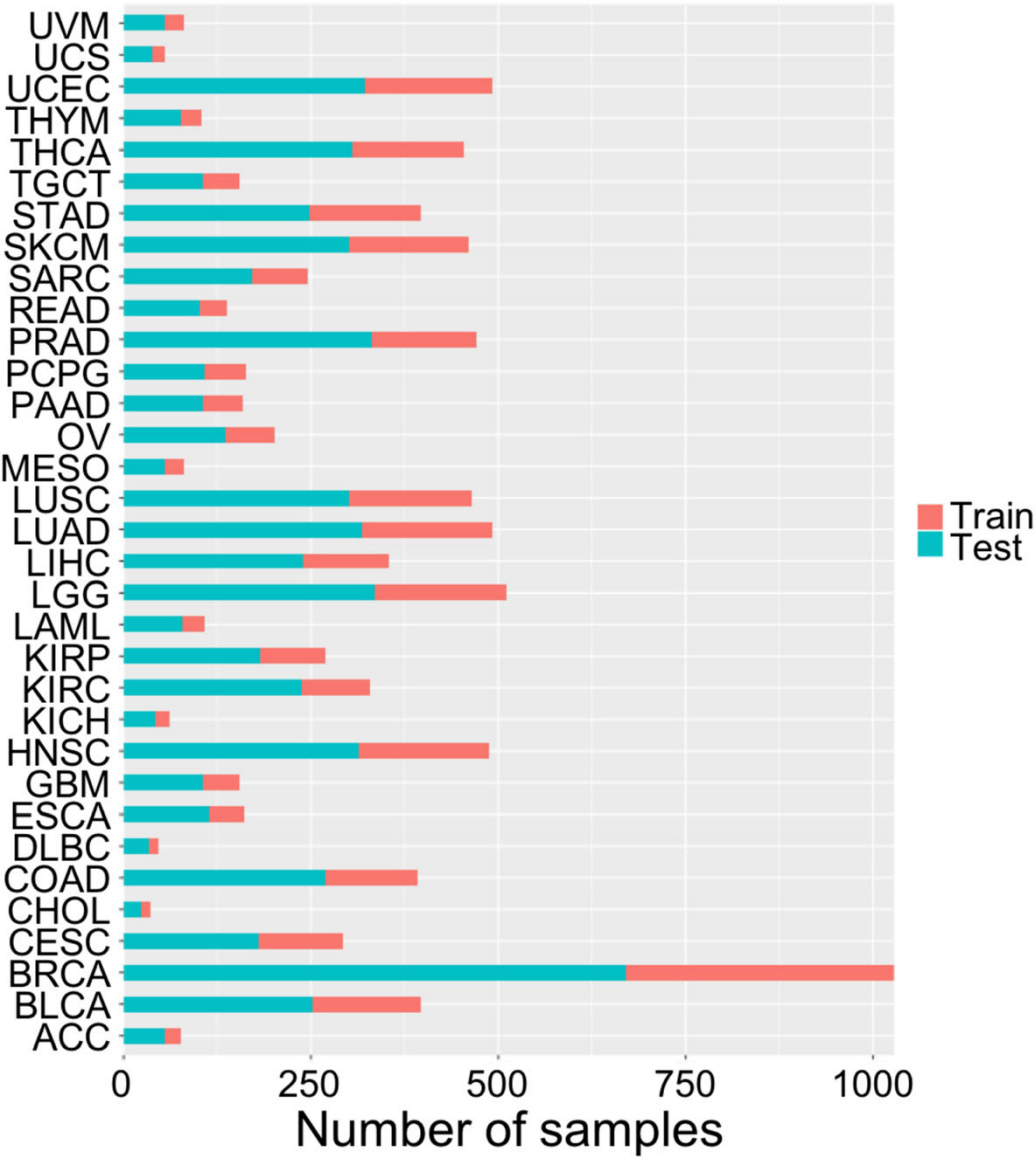
Graphic illustration of the numbers of training and testing samples for each tumor type used in the analyses for the 33 TCGA tumor samples. For names associated with tumor-type codes, see Additional file 2: Table S1

We obtained 9318 unique biological samples (Additional file 2: Table S1) after matching expression data with tumor purity estimates. We randomly divided the 9318 samples into 2/3 for training (6214 samples) and 1/3 for testing (3104 samples) (Fig. 1).

For an independent test dataset, we aggregated single-cell RNA-seq (scRNA-seq) expression data (GSE118390) from patients with triple-negative breast cancer (TNBC) [6] to obtain the “bulk” RNA-seq gene expression data. The original authors carried out the control analysis and included 1189 cells and 13,280 genes after filtering. The data for cancer cell proportions in those samples (Additional file 3: Table S2) were kindly provided to us by the authors and shown in Fig. 1 of [6].

## Results

### Tuning parameters

After testing a total of 1486 parameter combinations on the training data using a 10-fold cross-validation procedure, we chose the parameter set with the best cross-validation performance to use for all subsequent analyses unless stated otherwise. The cross-validation performance of each parameter combination for pan-cancer purity prediction is shown in Additional file 4: Figure S2. There was a tradeoff between number of trees and learning rate, i.e., smaller learning rate required more trees. Therefore, we fixed the maximum number of trees to be a large number (up to 5000) with reasonable computational time and adopted an early stopping rule: stopping when training performance for the dataset did not improve in five additional trees. The best parameter set for all tumor types combined was: learning rate of 0.05, maximum tree depth of 4, minimum leaf weight of 1, 65% of genes used to grow each tree, and 85% of samples used to grow each tree.

### Performance on pan-cancer tumor purity prediction considering all genes

For each tumor type, we built 1000 models through 100 repetitions of 10-fold cross-validation. We ran each model with the same selected tuning parameter set. Each individual model consists of a sequence of trees. Although the average training performance of these individual models was nearly perfect [near perfect correlation between observed and predicted values and low root mean squared error (RMSE)], overtraining was not a major concern as both cross-validation and test performances were also high (Additional file 5: Table S3A).

**Fig. 2:**
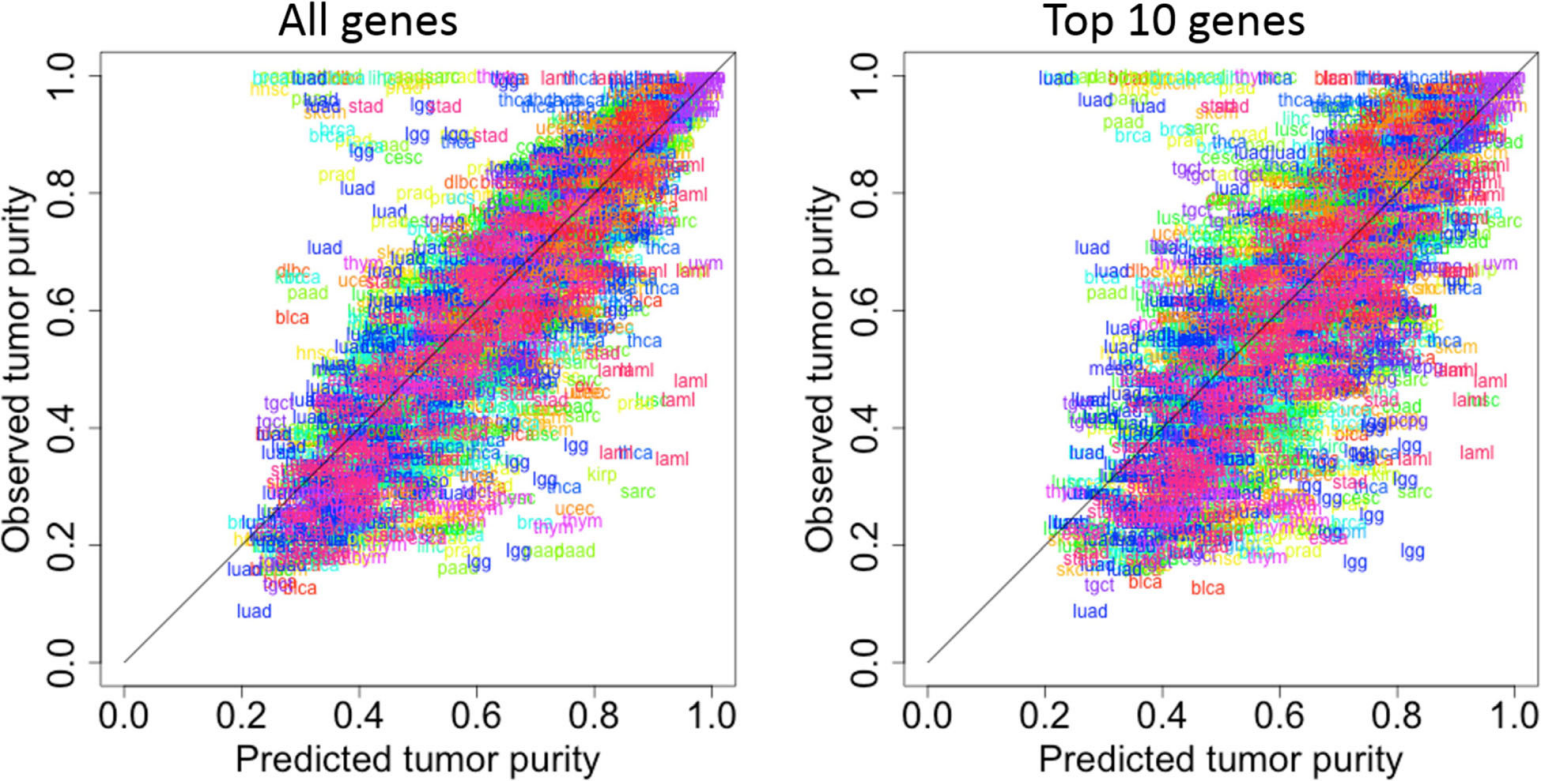
Scatterplots of pan-cancer tumor purity values for the test samples: predicted values by XGBoost versus the observed values from ABSOLUTE. **a**, using all genes as the predictors; and **b**, using only the ten marker genes as predictors. Each label represents a test sample and each tumor type is colored differently

To construct our final predictor for the test samples, we averaged the predicted tumor purity values from the 1000 predictions for each of those samples. The Pearson correlation coefficient and RMSE between the final predicted and observed tumor purity values for the test samples were 0.795 (Fig. 2a) and 0.129, respectively.

To see if this observed prediction performance could have been achieved by chance, we applied our procedure to putatively null data sets generated by permutation (Additional file 6: Text - Permutation test). We showed that the models by XGBoost are meaningful and the good performance is unlikely to be attributable to chance (empirical *P* < 0.004).

### Top-ranked genes for pan-cancer tumor purity prediction

**Fig. 3:**
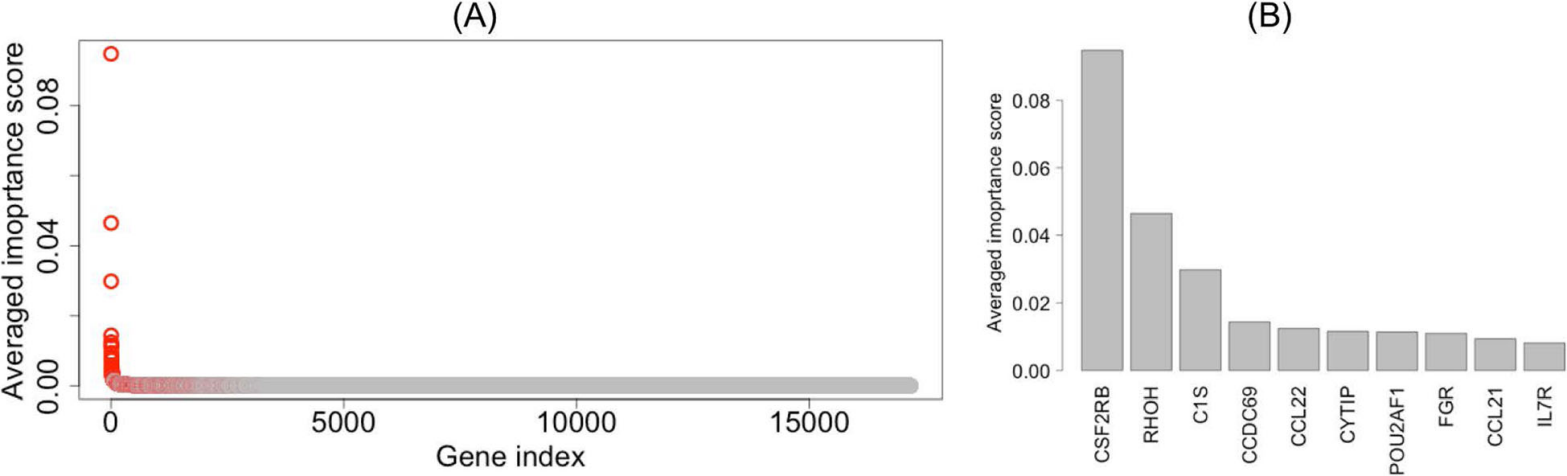
Plot of the average importance score versus rank of the gene by average importance score (gene index). **a**, Genes shown in red have non-zero average importance scores across all 1000 models. **b**, zoom in of the top 10 genes

Each of the 1000 models provided an importance score for each gene from up to 5000 boosts (see **Methods**). We averaged the 1000 scores for each gene to obtain the average score for the gene. When ordered, the overall importance score decayed quickly (Fig. 3a). Among the 17,170 genes, 731 had non-zero importance scores in all 1000 models. We re-ranked these genes based on the median value instead of the mean of the 1000 importance scores for each gene. The ten top-ranked genes remained the same for both mean- and median-based ranking. The top ten genes (Fig. 3b) were *CSF2RB*, *RHOH*, *C1S*, *CCDC69*, *CCL22*, *CYTIP*, *POU2AF1*, *FGR*, *CCL21*, and *IL7R* (Additional file 7: Table S4).

### Performance on pan-cancer tumor purity prediction using only the ten marker genes

To see if the ten marker genes could serve as “universal” markers for tumor purity prediction using RNA-seq gene expression data, we repeated the above analysis using only the ten marker genes. First, we selected tuning parameters tailored to these ten marker genes using the procedure described (see Methods: Tuning parameters). With this new set of tuning parameters, we again carried out 100 repetitions of 10-fold cross-validation with the same training samples as before. Lastly, we applied the 1000 resulting models to predict tumor purity values in the same test samples. To our surprise, the ten genes did reasonably well. The average performance of these individual models with only ten marker genes as predictors was comparable, though slightly degraded, compared to using all genes as predictors (Additional file 5: Table S3b).

For the performance of our overall predictor based on averaging predictions from 1000 models, the Pearson correlation and RMSE between the final predicted tumor purity values and observed tumor purity values for the test samples were 0.719 (Fig. 2b) and 0.146, respectively. Thus, performance using only these ten marker genes suffers only slightly compared to performance using all genes.

### Performance on individual tumor type purity prediction using only the ten marker genes

**Fig. 4:**
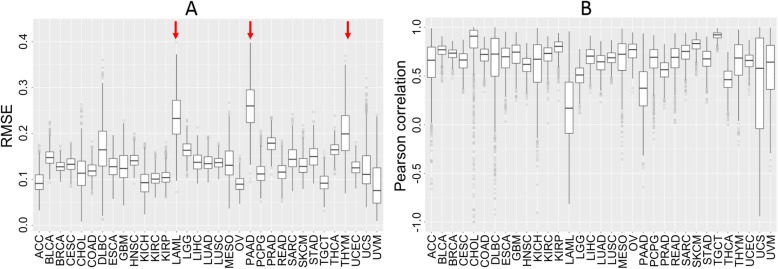
Box plots of cross-validation performance using the ten marker genes for each of the 33 tumor types: **a**, RMSE; and **b**, Pearson correlation. The white box extends from the 25th to the 75th percentiles with the median at the horizontal line. Based on median RMSE, the three tumor types (LAML, PAAD, and THYM) performed relatively poorly compared to others (red arrows)

Above, we showed that the ten genes performed well on pan-cancer (combined) tumor purity prediction. To see if the ten marker genes could also perform well for individual tumor types, we carried out training and cross-validation for each of the 33 tumor types, separately. Specifically, for each tumor type, we carried out the same 100 repetitions of 10-fold cross-validation using only the ten marker genes. However, we did not create a separate testing set as we did for pan-cancer purity prediction because several tumor types have small sample sizes (< 100). We identified an optimal set of tuning parameters for each tumor type using the same approach (see Methods: Tuning parameters) as with all tumor types combined. Across the 33 tumor types, the predictive performance of the ten marker genes varied widely: the median Pearson correlation ranged from 0.17 to 0.92 whereas the RMSE ranged from 0.08 to 0.26 (Fig. 4).

Although the ten marker genes performed reasonably well for most tumor types, performance was poor for three tumor types (LAML, PAAD, and THYM) with high median RMSE. The poor performance for LAML is likely due to the fact that we limited the genes to the 10 markers, which may not be the optimal for those tumor types. LAML (acute myeloid leukemia) is a blood-borne cancer. We speculate that the models obtained from the largely solid tumors may not be appropriate for blood cancer. For PAAD and THYM, interestingly, two other methods (ESTIMATE [14] and CONSENSUS [15]) did not provide tumor purity estimates. For the 53 PAAD test samples, we computed both RMSE and correlations between XGBoost predicted tumor purity values and ABSOLUTE estimated tumor purity values and compared those from InfiniumPurify [12], a DNA methylation-based tumor purity prediction method. The predicted tumor purity values from XGBoost and InfiniumPurify both deviate from those from ABSOLUTE for some of the same samples (Additional file 8: Table S5A). For THYM, like XGBoost, there was little correlation between ABSOLUTE estimated tumor purity values and InfiniumPurify estimated tumor purity values (Additional file 8: Table S5B).

### Comparison with ESTIMATE

In this comparison, we only considered the 2359 test set samples that were common between our 3104 test samples and all samples with purity data from ESTIMATE [14]. XGBoost outperformed ESTIMATE with a smaller RMSE (0.12 using all genes and 0.14 using the ten marker genes compared to 0.25 from ESTIMATE) and higher correlations (0.82 using all genes and 0.73 using the ten marker genes compared to 0.61 from ESTIMATE). The results are summarized in Additional file 9: Table S6. Moreover, ESTIMATE used 141 stromal and 141 immune marker genes whereas XGBoost used as few as 10 with better performance.

### Comparison with random Forest

For XGBoost, we did not specifically search the hyperparameter space for the optimal hyper-parameter set for RF. Instead, we used the best hyper-parameter set we obtained from our XGBoost analysis as the candidates for the best parameter for RF and further carried out fine-grid search around the candidate set (Additional file 10: Table S7). We chose the hyperparameters (1000 trees, 100% samples per tree and 60% features per split), which gave rise to the lowest out-of-bag error (an indicator for generalization error) as the best hyper-parameter set for RF.

RF performed comparably for the test samples for all parameter combinations tested. The RMSE, Pearson correlation, and Spearman correlation between the predicted and observed tumor purity values for the test samples were in the ranges of [0.150–0.151], [0.91–0.98], and [0.777–0.783], respectively. The best result is provided in Table 1.

**Table 1:** Performance comparisons between XGBoost and Random Forrest on the pan-cancer dataset

Method	Number of genes used	RMSE	Correlation coefficient	
			Pearson	Spearman
Random Forrest	all	0.151	0.940	0.780
XGBoost	all	0.124	0.806	0.813
	5	0.150	0.701	0.708
	**10**	**0.146**	**0.719**	**0.726**
	20	0.139	0.751	0.756
	100	0.130	0.787	0.794
	200	0.128	0.792	0.799
	300	0.126	0.800	0.807

XGBoost performed better than RF in terms of RMSE and Spearman correlation (Table 1) but worse in terms of Pearson correlation, which is subject to more influence by outliers than Spearman correlation. We believe that recursively fitting on the regression residues in XGBoost [30] contributed to the better performance. Moreover, it is worth noting that the XGBoost achieved similar RMSE using only five genes/features compared to all genes by RF (Table 1). We did not carry out feature selection using RF as it is computationally prohibitive for this dataset.

### Performance on the independent TNBC dataset using all genes

**Fig. 5:**
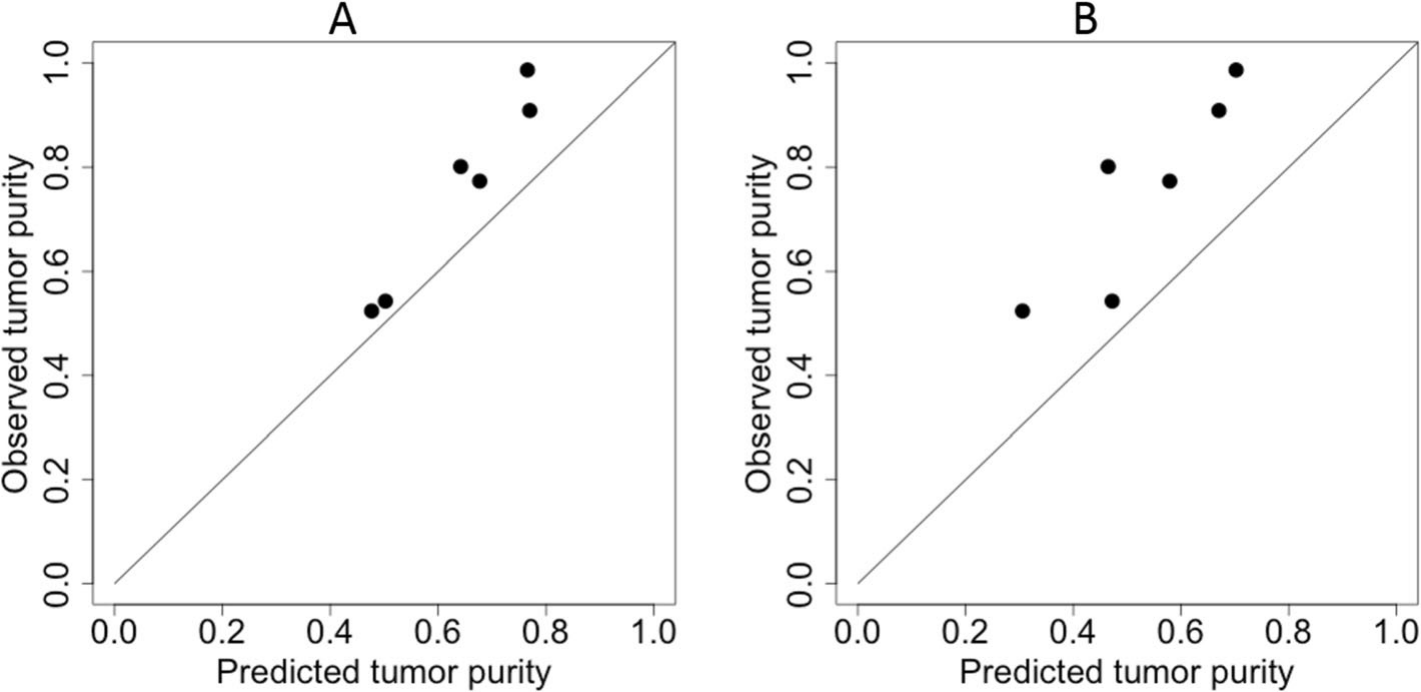
Scatterplots of the XGBoost predicted tumor purity values versus the observed tumor purity values constructed from single-cell RNA-seq experiments for six independent TNBC samples. **a**. using all genes as the predictors; and **b**, using only the 10 marker genes as the predictors

Using the 134 TNBC RNA-seq samples from TCGA as the training samples, we also carried out the same 100 repetitions of 10-fold cross-validation. Predictions from the resulting 1000 models were subsequently averaged to predict tumor purity values for the six independent TNBC samples (not used in training) for which the “bulk” RNA-seq data were aggregated from scRNA-seq data [6]. Average cross-validation performance of the individual models was reasonably good, but we were predicting only six test samples (Additional file 11: Table S8A). For the overall predictor, the correlation coefficient between the experimentally obtained tumor purity values and our XGBoost predicted tumor purity values was high (ρ = 0.98, Pearson correlation) (Fig. 5a) and the RMSE was low (0.133). This good performance by XGBoost happened even though the model was trained on ABSOLUTE purity estimates but purity estimates in the test samples were based on cell types of the individual cells from scRNA-seq data sets.

### Performance on the independent TNBC dataset using only the ten marker genes

To see if the ten putative marker genes could also accurately predict tumor purity of the six independent samples, we repeated our entire analysis procedure (tuning parameter optimization; 100 repetitions of 10-fold cross-validation; averaging the 1000 predictions for each test sample) using the expression data of only the ten genes in the 134 training samples from TCGA. Average cross-validation performance of the individual models was comparable but using 10 genes as predictors was worse compared to using all genes (Additional file 11: Table S8b). The correlation between the final predicted and experimentally obtained tumor purity values remained high (*ρ* = 0.88) (Fig. 5b), though the RMSE (0.239) was nearly twice as large as it was when using all genes.

## Discussion

We applied XGBoost, a machine learning algorithm, to genome-wide RNA-seq data to unbiasedly select a subset of genes whose expression values could predict tumor purity obtained from ABSOLUTE analysis using copy number variation [8]. We combined 9318 TCGA tumor samples from 33 tumor types, carried out pan-cancer tumor purity prediction, and evaluated the quality of predictions through 100 repetitions of 10-fold cross-validation (Additional file 6: Text - Testing for convergence). Our final predictor was the average of 1000 predictions for independent test samples. We used all genes as well as a selected subset of ten marker genes in our predictions. XGBoost performed well and the correlation between the predicted and observed tumor purity values was generally high with low RMSE.

XGBoost provides an importance score for each gene from each model reflecting how useful or important the gene was to the model’s prediction. We wondered if the top-ranked ten genes could be used as a “universal” biomarker set for tumor purity prediction. To test this idea, we carried out three separate analyses using only the expression data of the ten marker genes. First, we carried out pan-cancer (all 33 tumor types combined) tumor purity prediction through model training, cross-validation, and testing. Second, we carried out tumor purity prediction for individual tumor types through model training and cross-validation. Lastly, we trained XGBoost models on 134 TCGA triple-negative breast cancer RNA-seq tumor samples and used the resulting models to predict tumor purity of six independent samples that were not from TCGA and not used in model training. In these analyses, we showed that the correlation between the predicted and observed tumor purity values was relatively high and the RMSE was generally small except for three tumor types (LAML, PAAD, and THYM). Therefore, we suggest that the ten-gene set could serve as a biomarker for tumor purity prediction using gene expression data.

We speculate that most of the ten marker genes are largely expressed by the tumor stroma, not the cancer cells. It is not clear if the expression of these genes in tumors merely reflects the amount of infiltrating immune cells or is indicative of some (unknown) fundamental biological processes. All genes, except for two (*C1S* and *CCDC69*), had high expression in either transformed lymphocytes, whole blood, or spleen (https://gtexportal.org), suggesting that these genes may be predominantly expressed by the infiltrating immune cells in the tumors. C1S was ubiquitously expressed in most organs except for the brain. C1S was also ubiquitously expressed in most cell lines with highest expression in transformed fibroblasts, an important component of the tumor microenvironment. However, immunostaining of tumor tissue samples showed that all malignant cells were negative (https://www.proteinatlas.org/), suggesting that the observed *C1S* expression in tumor samples may also largely come from the tumor stroma. Like *C1S*, *CCDC69* was also ubiquitously expressed except in the brain. However, unlike *C1S*, malignant cells also showed moderate to strong cytoplasmic staining in tumor samples for nearly all TCGA tumor types. Interestingly, the expression levels of *CCDC69* in TCGA tumor samples were negatively correlated with the tumor purity for the same samples for nearly all TCGA tumor types (data not shown), suggesting that the observed *CCDC69* expression in those tumor samples may also largely come from the tumor stroma. Taken together, these results appear to suggest that these genes are largely expressed in the tumor stroma and that may explain why they were selected as the most important genes for tumor purity prediction.

Many methods have been proposed for tumor purity prediction, such as CONSENSUS [15] and ESTIMATE [14]. Other methods for tumor purity predictions consider methylation data [11–13] and copy number variation [8, 36, 37]. Most non-genomic (e.g., transcriptome- or methylome-based) purity prediction methods consider a subset of preselected stromal-cell-expressed genes or stromal-cell-specific methylation loci as predictors. ESTIMATE involves a set of 141 “universal” stromal genes selected using a sophisticated computational scheme [14]. In our approach, XGBoost considered all genes. Furthermore, our marker genes were not preselected, but identified by XGBoost.

Tumor purity is inversely correlated with the expression of genes active in stromal gene expression. We found that the expression levels of most predictive genes were negatively correlated with tumor purity. Boost also identified some genes as predictive whose expression levels were positively correlated with tumor purity (data not shown), suggesting that those genes were expressed primarily by cancer cells in the tumor samples. We plan to use XGBoost to systematically analyze each tumor type separately. We envision that there exist common and unique gene sets that are predictive of tumor purity among different tumor types. We believe that those common and unique genes might be reflective of the commonality and uniqueness of their respective tumor microenvironments and that identifying them might shed light on barriers to the efficacy of immunotherapy with different tumor types [38].

Our analysis required training data with both tumor purity estimates and gene expression data. To our knowledge, only TCGA has produced large datasets with both attributes. This makes independent validation of our models challenging. Single-cell experiments can estimate cell populations in a tissue sample. However, most of the single-cell experiments do not care about RNA-seq expression in bulk samples and the number of tissue samples considered in a single-cell experiment is typically small (e.g., under 10) due to high sequencing cost. Nonetheless, we could infer the “bulk” RNA-seq expression of a tissue sample from the expression data of individual cells in the tissue. Using scRNA-seq data from tissue samples from six TNBC patients, we showed that XGBoost trained on TCGA RNA-seq samples can predict cancer cell proportions in these independent test samples with high correlation using only the 10 marker genes.

The approach that we outlined uses XGBoost to derive predictive biomarkers will be applicable to expression data from any platform like microarrays (see Additional file 6: Text - Test on microarray data and Additional file 12: Figure S3), but the quality of the predictions would certainly depend on how well the data from a given platform reflected the underlying biological reality. The XGBoost algorithm should work well regardless of preprocessing or normalization steps [33]. If the data from different platforms provided comparably accurate reflections of the underlying reality, we would expect the identified biomarkers to serve well, regardless of platform. On the other hand, the exact predictive rule that we derived using RNA-seq data from TCGA will not necessarily transfer to other platforms or other scaling or normalization on the same platform. Our predictive rule relies on regression trees where the predictors in each regression are expression levels. To the extent that expression levels from different platforms are inherently on different scales or have been normalized differently, the estimated coefficients in the component regression models derived from one platform will differ from the corresponding estimated coefficients derived from another platform. Consequently, the exact predictive rule, which involves specific estimated coefficients, derived from data of one platform may not perform well on data from a different platform.

The robustness of gradient boosting machines against small perturbations has been documented [39]. We believe that the TCGA data that we used was appropriately normalized. Nonetheless, outliers can be problematic. Robust methods for dealing with data with outliers have been developed and are thoroughly reviewed [40].

Like many other methods that minimize the L2 loss function, XGBoost would not be robust to outliers/contamination in the response variable. This means that the fidelity of our predictive models depends on the quality of the tumor purity data. For three TCGA tumor types (LAML, PAAD, and THYM), XGBoost performed poorly. This poor performance was also confirmed by an independent method, InfiniumPurify [12], which uses DNA methylation data from largely the same patients for tumor purity prediction. For these three tumor types, the models may be mis-specified or the tumor purity values may be “outliers”. Given this finding, we would not recommend using our models to predict tumor purity predictions for those three tumor types.

There are other ensemble learning algorithms applicable to our problem. XGBoost has some advantages, especially, its low computational time complexity and high performance [31]. The XGBoost software is optimized for large-scale machine learning problems on high performance computers. This efficiency is especially needed for our dataset which consisted of ~ 20,000 genes/variables and ~ 10,000 samples.

Finally, we acknowledge that our ten marker genes may not be optimal for all tumor types. It is likely that tumor-type specific predictive models may perform better than models derived from pan-tumors. We were surprised that such a “universal” (although imperfect) gene set could be found, suggesting that expression levels of some immune genes in solid tumors might be indicative of the amount of immune cell infiltration in tumors. Also, our external validation of the ten marker genes as predictive biomarkers was limited by small sample size with only six samples. This is typical of current single cell studies due to high cost. As the single cell sequencing technologies improve further, analysis of a larger number of tissue samples may likely be routine. Until then, we believe purity prediction using marker genes or methylation sites remains useful.

In summary, we have demonstrated that XGBoost can identify a subset of genes whose expression levels could predict tumor purity. We propose that the expression levels of the ten genes may serve as biomarkers for tumor purity estimation.

## Methods

### Framework

**Fig. 6:**
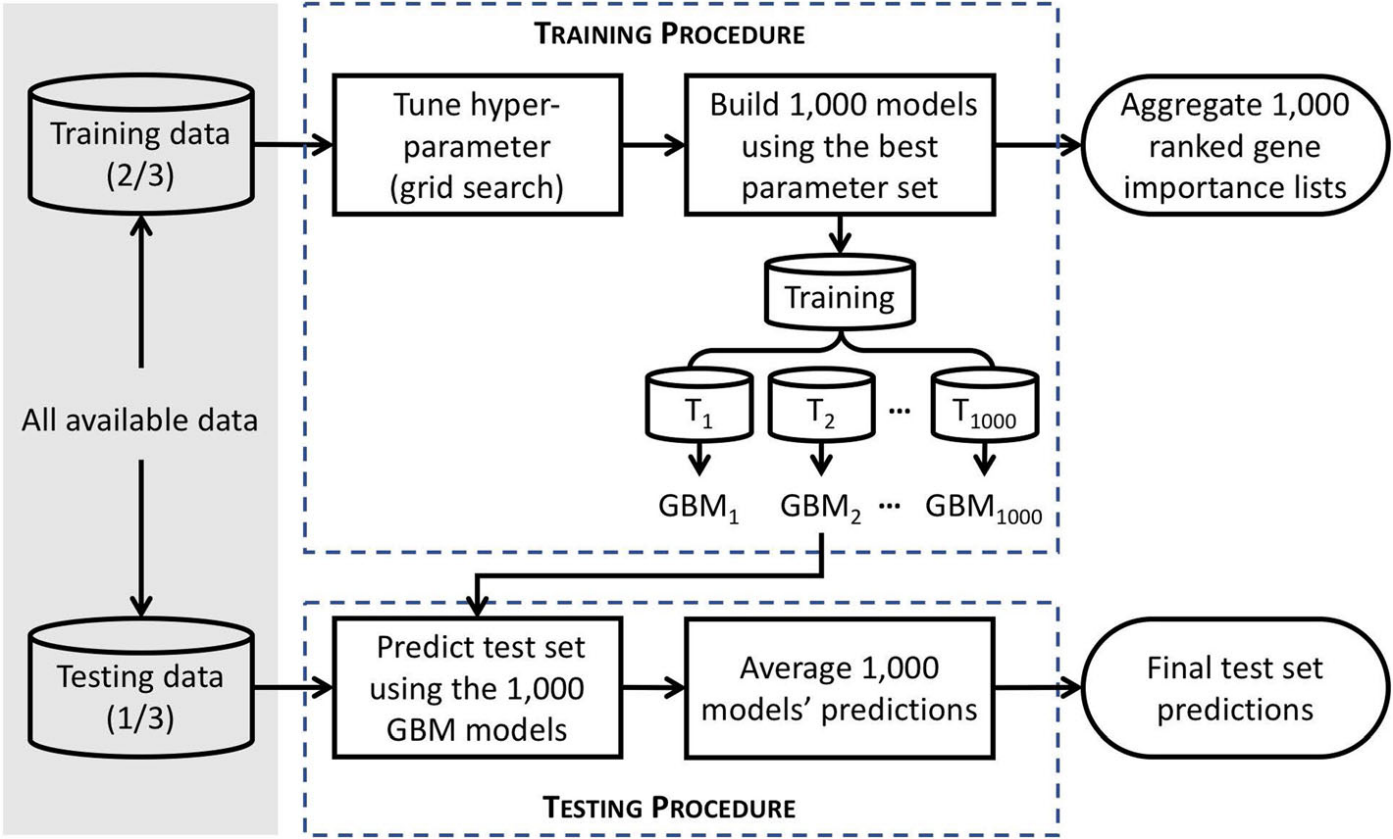
A schematic of the XGBoost workflow. The shaded area indicates the data and its partitioning. The boxes inside the dashed lines depict training and testing procedures where T stands for tree and GBM stands for gradient boosting machine. The two oval boxes on the right denote the outputs from XGBoost. A tree-representation of the training and testing procedures is provided in Additional file 13: Figure S4

We built an ensemble of stochastic gradient boosted tree models using the training data to predict tumor purity in the test set samples (Fig. 6 and Additional file 13: Figure S4). Specifically, we carried out 100 repetitions of 10-fold cross-validation within the original training data. Each repetition was created by randomly shuffling the order of the training set. Then, for each 10-fold cross-validation, 10% of the samples were sequentially set aside as validation samples and the remaining 90% of the samples used as training samples. This procedure created 100 × 10 = 1000 training-validation partitions of the original training samples. Based on RMSE, 1000 models appear to be adequate (Additional file 14: Figure S5). We fit the XGBoost (R package: version 0.82.1; https://cran.r-project.org/web/packages/xgboost/) models to each training subsample and used the resulting fitted models to predict the tumor purity values of the corresponding validation subsamples and again for the original test data (3104 samples). We used the average of 1000 predictions for each test sample as its final tumor purity prediction. Our reasons of using ensemble of tree ensembles are twofold. First, we could boost the prediction performance by leveraging a model averaging approach. Secondly, since we sought to avoid overfitting by using only a random subset of genes to grow each tree, we could by chance rank an important predictor/gene low. To ensure that we ranked genes appropriately, we repeated the procedure with slightly different training samples many times.

### Tuning parameters

XGBoost employs eight tuning parameters (Additional file 15: Table S9) to control “bias-variance” trade-offs. Complicated models (e.g., many boosted trees in a sequence) may fit the training data well but not the testing data, a situation called “overfitting.” XGBoost provides two general ways to avoid overfitting. First, one could adjust model complexity by the changing values of tuning parameters: maximum tree depth, minimum leaf weight, and minimum split gain. The number of trees and the tree depth determine the final tree’s structure and complexity. Because each new tree in sequence tries to correct mistakes made by previous trees, shallow trees with a depth of 4–6 are often preferred [30]. The second way is to add randomness to make training more robust to noise. Randomness can be adjusted by setting the sub-sampling rate at each sequential tree and/or by using a subset of randomly selected features for splitting. The model’s learning rate, another important tuning parameter, determines how much each tree contributes to the overall model. A low learning rate will increase the number of trees in a sequence and should result in better performance. The tradeoff is that it will increase computational cost. The final predictive model is a linear combination of all trees in the sequence with their contributions weighted by the learning rate.

To identify the best combination of tuning parameters, we carried out a 10-fold cross-validation on the training data (6214 samples) for each of 1486 possible tuning parameter combinations. This step took about one week to complete on a single server (Intel processors, 64 cores, 2.30 GHz CPU). We used the parameter combination with the best cross-validation performance to train the final model using the training data.

### Feature importance score

XGBoost automatically provides estimates of feature importance from a trained predictive model. “Feature importance score” refers to a score indicating how useful or important the feature (in this case, a gene) was to the model’s prediction. Here, the importance score is calculated for a given gene for a single tree by summing the amount that each split point involving that gene improved the performance measure, weighted by the number of observations contributing at each split [31]. Denote the importance score for gene *g* in an individual tree *t* by *S*_*gt*_. Within a single model composed of a sequence of trees, the importance score for a particular gene *g*^∗^ in a model is the sum of all tree-specific importance scores for that gene over all trees in the model divided by the sum of all importance scores over all genes and all trees in the model, namely, ${S}_{g^{\ast}\bullet }=\left({\sum}_t^T{S}_{g^{\ast }t}\right)/\left({\sum}_t^T{\sum}_g^G{S}_{gt}\right)$. We picked the genes with non-zero importance scores for all 1000 models (each fitted to a distinct training-testing partition of the original data; see below). For each model, we got a ranked list of genes based on their variable importance scores. We aggregated 1000 ranked lists of genes by ranking genes based on their median rank across the 1000 models.

### Comparisons with ESTIMATE and Random Forests

The tumor purity values from ESTIMATE [14] were downloaded from ref. [14]. For the Random Forest analysis, we used the MATLAB built-in function (TreeBagger). We followed the same training-testing procedure as we did for XGBoost.

### Performance evaluation

To evaluate performance, we compared the predicted tumor purity values with the observed tumor purity values. For each model, we computed both the RMSE and Pearson correlation (ρ) between the predicted tumor purity values and the ABSOLUTE estimated purity values as performance measures. To summarize the performance of individual XGBoost models, we computed both mean and standard error and median for each measure (both correlation and RMSE) across the 1000 training-validation partitions and testing data. To create the final predicted value for each test sample, we also averaged the 1000 predicted tumor purity values for the sample. The averaged predicted purity value can be viewed as the predicted value by bagging ensembles (1000 in our case). Prediction using bagging ensembles performs well [41] as shown in Random Forests [42]. Throughout the remaining manuscript, we referred to this predicted value by bagging ensembles as the (final) single predicted value and we report its RMSE and correlation with ABSOLUTE tumor purity as measures of overall performance.

Let *ŷ* denote the predicted tumor purity value, *y* be the tumor purity value estimated by ABSOLUTE (observed) and *N* be the sample size.

$$ RMSE\left(y,\hat{y}\right)=\sqrt{\frac{1}{N}\sum \limits_i^N{\left({y}_i-{\hat{y}}_i\right)}^2} $$

### Obtaining “bulk” RNA-seq data from scRNA-seq data

For each of the six TNBC scRNA-seq datasets, we summed the raw expression counts for each gene across all cells in the sample to obtain the raw gene-specific expression count for the “bulk” sample. This procedure resulted in six “bulk” expression profiles, one for each of the six samples. Next, we extracted the 134 TNBC RNA-seq samples from TCGA breast cancer RNA-seq samples. The two datasets were then merged using the common genes (15,076). Next, we normalized all 140 samples in the combined dataset using the median of the medians of expression values of the 134 TCGA samples. Specifically, we calculated median expression for each sample and then centered all the data on the median of those medians. Finally, we log_2_-transformed the normalized counts (log_2_(count+ 1)). The 134 TCGA samples were used to train our model, and the resultant model was then applied to predict tumor purity values for the six independent “bulk” samples.

### R code and test data

We have included the R source code, a demo dataset (TCGA triple negative breast cancer and an independent bulk RNA-seq data from single cell sequencing), and a brief documentation on Github (https://github.com/yuanyuanli66/gbm.ensemble). The code allows users to build their own models using the TCGA RNA-seq data (not provided) for tumor purity prediction on their own expression data. Because of the size of the TGCA data, we did not include the RNA-seq data for all tumor types in the package. Such data can be downloaded from the Pan-Cancer Atlas Publication website (https://gdc.cancer.gov/about-data/publications/pancanatlas).

## Supplementary information

**Additional file 1: Figure S1.** Box plots of tumor purity estimates by ABSOLUTE in original scale [0–1] for each tumor type.

**Additional file 2: Table S1.** Number of samples with both ABSOLUTE tumor purity data and RNA-seq gene expression data for each of the 33 tumor types.

**Additional file 3: Table S2.** Cancer cell proportion in tumor samples from triple negative breast cancer patients.

**Additional file 4: Figure S2.** Plots of cross-validation performances of various tuning parameter combinations: (top) RMSE; and (bottom) Pearson correlation.

**Additional file 5: Table S3.** Summary of pan-cancer tumor purity prediction performance of individual XGBoost models across 1000 separate training-validation partitions (A) using all genes; (B) using only the 10 marker genes.

**Additional file 6: Text.** Testing for convergence, permutation test, and test on microarray data.

**Additional file 7: Table S4.** Annotation of the ten marker genes.

**Additional file 8: Table S4.** Annotation of the ten marker genes.

**Additional file 9: Table S6.** Performance comparison between ESTIMATE and XGBoost for the test set samples

**Additional file 10: Table S7.** Hyper-parameter search for Random Forrest models.

**Additional file 11: Table S8.** Summary of TNBC sample purity prediction performance of individual XGBoost models across 1000 training-validation partitions. (A) using all genes; (B) using only the ten marker genes.

**Additional file 12: Figure S3.** Scatter plot of XGBoost predicted and ABSOLUTE estimated tumor purity values for the 162 test set microarray samples.

**Additional file 13: Figure S4.** A schematic of our performance evaluation strategy.

**Additional file 14: Figure S5.** Box plots of RMSE of the predicted versus the observed tumor purity values for various number of models for the pan-cancer data.

**Additional file 15: Table S9.** XGBoost tuning parameters and those selected as the optimal set.

## Data Availability

The datasets analyzed in this study were downloaded from the Pan-Cancer Atlas Publication website (https://gdc.cancer.gov/about-data/publications/pancanatlas).

## Abbreviations

C1S: Complement C1s
CART: Classification And Regression Tree
CCDC69: Coiled-coil domain containing 69
CCL21: C-C motif chemokine ligand 21
CCL22: C-C motif chemokine ligand 22
CSF2RB: Colony stimulating factor 2 receptor beta common subunit
CYTIP: Cytohesin 1 interacting protein
FGR: FGR proto-oncogene, src family tyrosine kinase
IL7R: Interleukin 7 receptor
LAML: Acute Myeloid Leukemia
PAAD: Pancreatic adenocarcinoma
POU2AF1: POU class 2 associating factor 1
RHOH: Ras homolog family member H
RMSE: Root Mean Squared Error
TCGA: The Cancer Genome Atlas
THYM: Thymoma
TNBC: Triple-Negative Breast Cancer
XGBoost: EXtreme Gradient Boosting
