# Supplementary material for: Putative biomarkers for predicting tumor sample purity based on gene expression data
Source: BMC Genomics. 2019 Dec 27;20:1021. doi: 10.1186/s12864-019-6412-8 (PMC6933652; doi:10.1186/s12864-019-6412-8)
Supplement: Supplementary file 1 — Additional file 1: Figure S1. Box plots of tumor purity estimates by ABSOLUTE in original scale [0–1] for each tumor type. [file 12864_2019_6412_MOESM1_ESM.docx]

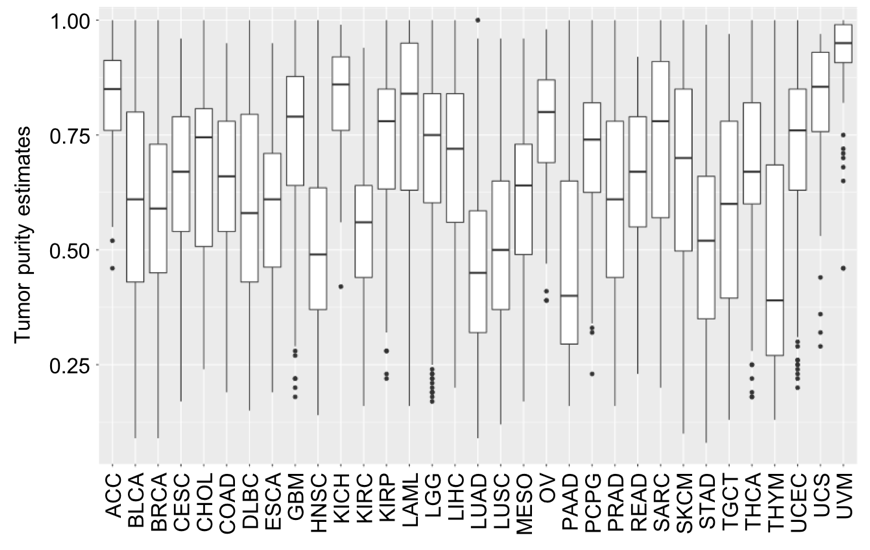


**Figure S1**. Box plots of tumor purity estimates by ABSOLUTE in original scale [0-1] for each tumor type. The white box extends from the 25^th^ to the 75^th^ percentiles with the median at horizontal line.
