# Supplementary material for: Putative biomarkers for predicting tumor sample purity based on gene expression data
Source: BMC Genomics. 2019 Dec 27;20:1021. doi: 10.1186/s12864-019-6412-8 (PMC6933652; doi:10.1186/s12864-019-6412-8)
Supplement: Supplementary file 2 — Additional file 2: Table S1. Number of samples with both ABSOLUTE tumor purity data and RNA-seq gene expression data for each of the 33 tumor types. [file 12864_2019_6412_MOESM2_ESM.docx]

**Table S1**. Number of samples with both ABSOLUTE tumor purity data and RNA-seq gene expression data for each of the 33 tumor types. The number used in testing is listed in parentheses

| **CODE** | **Name** | **Number of samples (number in testing)** |
| --- | --- | --- |
| ACC | Adrenocortical carcinoma | 76 (22) |
| BLCA | Bladder urothelial carcinoma | 397 (144) |
| BRCA | Breast invasive carcinoma | 1,028 (357) |
| CESC | Cervical squamous cell carcinoma and endocervical adenocarcinoma | 293 (113) |
| CHOL | Cholangiocarcinoma | 36 (12) |
| COAD | Colon adenocarcinoma | 393 (124) |
| DLBC | Lymphoid Neoplasm Diffuse Large B-cell Lymphoma | 47 (14) |
| ESCA | Esophageal carcinoma | 162 (48) |
| GBM | Glioblastoma multiforme | 154 (48) |
| HNSC | Head and neck squamous cell carcinoma | 487 (174) |
| KICH | Kidney Chromophobe | 61 (18) |
| KIRC | Kidney renal clear cell carcinoma | 328 (90) |
| KIRP | Kidney renal papillary cell carcinoma | 270 (88) |
| LAML | Acute Myeloid Leukemia | 109 (31) |
| LGG | Brain Lower Grade Glioma | 510 (175) |
| LIHC | Liver hepatocellular carcinoma | 355 (116) |
| LUAD | Lung adenocarcinoma | 491 (173) |
| LUSC | Lung squamous cell carcinoma | 465 (165) |
| MESO | Mesothelioma | 81 (27) |
| OV | Ovarian serous cystadenocarcinoma | 201 (65) |
| PAAD | Pancreatic adenocarcinoma | 159 (53) |
| PCPG | Pheochromocytoma and Paraganglioma | 164 (55) |
| PRAD | Prostate adenocarcinoma | 470 (139) |
| READ | Rectum adenocarcinoma | 138 (36) |
| SARC | Sarcoma | 246 (74) |
| SKCM | Skin Cutaneous Melanoma | 460 (160) |
| STAD | Stomach adenocarcinoma | 397 (148) |
| TGCT | Testicular Germ Cell Tumors | 155 (49) |
| THCA | Thyroid carcinoma | 454 (148) |
| THYM | Thymoma | 155 (27) |
| UCEC | Uterine corpus endometrial carcinoma | 492 (169) |
| UCS | Uterine Carcinosarcoma | 56 (18) |
| UVM | Uveal Melanoma | 80 (24) |
| Total |  | 9,318 (3,104) |
