# Supplementary material for: Putative biomarkers for predicting tumor sample purity based on gene expression data
Source: BMC Genomics. 2019 Dec 27;20:1021. doi: 10.1186/s12864-019-6412-8 (PMC6933652; doi:10.1186/s12864-019-6412-8)
Supplement: Supplementary file 3 — Additional file 3: Table S2. Cancer cell proportion in tumor samples from triple negative breast cancer patients. [file 12864_2019_6412_MOESM3_ESM.docx]

**Table S2.** Cancer cell proportion in tumor samples from triple negative breast cancer patients^1^

| Patient ID | Cancer cell proportion |
| --- | --- |
| PT039 | 0.773 |
| PT058 | 0.524 |
| PT081 | 0.986 |
| PT084 | 0.543 |
| PT089 | 0.801 |
| PT126 | 0.909 |
