# Supplementary material for: Putative biomarkers for predicting tumor sample purity based on gene expression data
Source: BMC Genomics. 2019 Dec 27;20:1021. doi: 10.1186/s12864-019-6412-8 (PMC6933652; doi:10.1186/s12864-019-6412-8)
Supplement: Supplementary file 4 — Additional file 4: Figure S2. Plots of cross-validation performances of various tuning parameter combinations: (top) RMSE; and (bottom) Pearson correlation. [file 12864_2019_6412_MOESM4_ESM.docx]

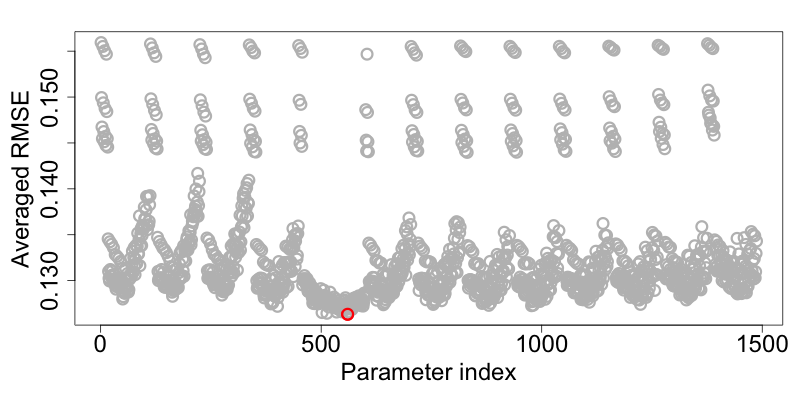


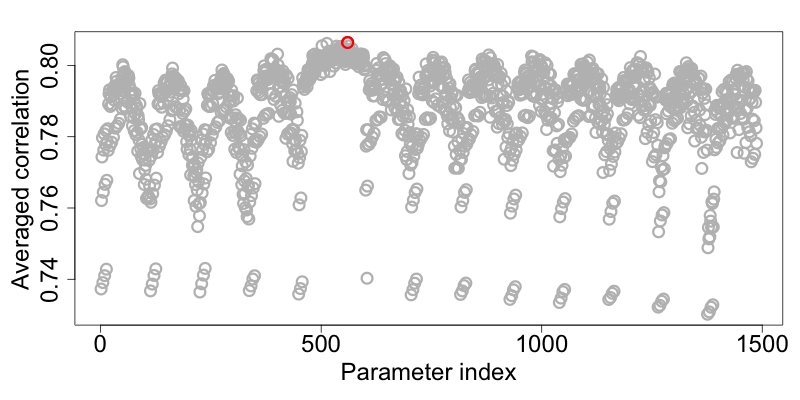


**Figure S2**. Plots of cross-validation performances of various tuning parameter combinations: (top) RMSE; and (bottom) Pearson correlation. Best parameter combination is marked in red.
