# Supplementary material for: Putative biomarkers for predicting tumor sample purity based on gene expression data
Source: BMC Genomics. 2019 Dec 27;20:1021. doi: 10.1186/s12864-019-6412-8 (PMC6933652; doi:10.1186/s12864-019-6412-8)
Supplement: Supplementary file 5 — Additional file 5: Table S3. Summary of pan-cancer tumor purity prediction performance of individual XGBoost models across 1000 separate training-validation partitions (A) using all genes; (B) using only the 10 marker genes. [file 12864_2019_6412_MOESM5_ESM.docx]

**Table S3**. Summary of pan-cancer tumor purity prediction performance of individual XGBoost models across 1,000 separate training-validation partitions (A) using all genes; (B) using only the 10 marker genes

| Procedure | Mean (standard deviation) | | Median | |
| --- | --- | --- | --- | --- |
|  | RMSE | Pearson correlation | RMSE | Pearson correlation |
| 1. Using all genes as predictors | | | | |
| Training | 6.4e-5 (8.4e-7) | 1.0 (1.8e-9) | 0.00006 | 1.0 |
| Cross-validation | 0.127 (0.006) | 0.804 (0.02) | 0.127 | 0.804 |
| Testing | 0.128 (0.0007) | 0.795(0.002) | 0.128 | 0.794 |
|  | | | | |
| 1. Using only the 10 marker genes as predictors | | | | |
| Training | 0.122 (0.0006) | 0.822 (0.002) | 0.122 | 0.822 |
| Cross-validation | 0.149 (0.006) | 0.714 (0.024) | 0.149 | 0.715 |
| Testing | 0.146(0.0003) | 0.717(0.001) | 0.146 | 0.717 |
