# Supplementary material for: Putative biomarkers for predicting tumor sample purity based on gene expression data
Source: BMC Genomics. 2019 Dec 27;20:1021. doi: 10.1186/s12864-019-6412-8 (PMC6933652; doi:10.1186/s12864-019-6412-8)
Supplement: Supplementary file 6 — Additional file 6: Text. Testing for convergence, permutation test, and test on microarray data. [file 12864_2019_6412_MOESM6_ESM.docx]

**Testing for convergence**

In all analyses, we carried out 100 repetitions of 10-fold cross-validation within the original training data. This resulted in 1,000 XGBoost models. To see how performance for our dataset depended on the number of XGBoost models, we randomly selected 100, 250, 500, and 750 models from the 1,000 models and computed the RMSEs between the predicted and ABSOLUTE estimated tumor purity values. For each set number of models, we repeated the process 100 times randomly. Boxplots (Additional file 14: Figure S5) summarizing the results for the 100 RMSEs for each set number of models indicate that the median RMSE declines steadily up to 750 models, reaching there the RMSE corresponding to the 1,000 models that we used. This result confirmed that the 1,000 models are indeed enough.

**Permutation test**

To see if this observed prediction performance could have been achieved by chance, we applied our procedure to putatively null data sets generated by permutation. We used one tumor type of average sample size, LUAD (Lung Squamous Cell Carcinoma). We randomly shuffled the observed tumor purity values among the samples while keeping the expression data unchanged to generate a new pseudo-dataset. As with the actual data, we carried out 100 repetitions of 10-fold cross-validation for each pseudo-dataset and used each of the resulting models to predict the tumor purity values in the testing set. We repeated the entire procedure 250 times by generating 250 pseudo-datasets independently and obtained 250 performance evaluations. We found that the RMSE for the pseudo data ranged from 0.16 to 0.21 whereas the median RMSE for the original data was 0.10, smaller than the smallest value from the shuffled data, resulting an empirical *P* <0.004. Similarly, the Pearson correlation coefficient for the shuffled data ranged from -0.25 to 0.20 and the median correlation for the original data was 0.82 (data not shown), much larger than the largest value from the shuffled data (empirical *P* <0.004). Those results strongly suggest that the models by XGBoost are meaningful and the good performance is unlikely to be attributable to chance.

**Test on microarray data**

To see if our method works on microarray data, we applied XGBoost to the BRCA microarray dataset from TCGA. We downloaded the level 3 lowess normalized gene level data from <https://gdac.broadinstitute.org/>. The data were normalized by TCGA and we did not carry out any additional normalization. Among the 590 microarray samples, 491 had the ABSOLUTE estimated tumor purity data. We divided the 491 samples in to a training set (329, 2/3 of the samples) and a testing set (162, 1/3 of the samples). We repeated our entire analysis procedure (tuning parameter optimization; 100 repetitions of 10-fold cross-validation; averaging the 1000 predictions for each test sample). The Pearson and Spearman correlations between the XGBoost predicted and ABSOLUTE estimated tumor purity values for the test set samples were 0.65 and 0.68, respectively (Additional file 12: Figure S3), suggesting that XGBoost can also work with microarray data for tumor purity prediction.
