# Supplementary material for: Putative biomarkers for predicting tumor sample purity based on gene expression data
Source: BMC Genomics. 2019 Dec 27;20:1021. doi: 10.1186/s12864-019-6412-8 (PMC6933652; doi:10.1186/s12864-019-6412-8)
Supplement: Supplementary file 7 — Additional file 7: Table S4. Annotation of the ten marker genes. [file 12864_2019_6412_MOESM7_ESM.docx]

**Table S4**. Annotation of the ten marker genes

| Symbol | Description |
| --- | --- |
| *CSF2RB* | colony stimulating factor 2 receptor beta common subunit |
| *AMICA1* | junction adhesion molecule like |
| *RHOH* | ras homolog family member H |
| *C1S* | complement C1s |
| *CYTIP* | cytohesin 1 interacting protein |
| *CCDC69* | coiled-coil domain containing 69 |
| *CCL21* | C-C motif chemokine ligand 21 |
| *CCL19* | C-C motif chemokine ligand 19 |
| *IL7R* | interleukin 7 receptor |
| *CCL22* | C-C motif chemokine ligand 22 |
