# Supplementary material for: Putative biomarkers for predicting tumor sample purity based on gene expression data
Source: BMC Genomics. 2019 Dec 27;20:1021. doi: 10.1186/s12864-019-6412-8 (PMC6933652; doi:10.1186/s12864-019-6412-8)
Supplement: Supplementary file 8 — Additional file 8: Table S4. Annotation of the ten marker genes. [file 12864_2019_6412_MOESM8_ESM.docx]

**Table S5.** Performance of XGBoost and InfiniumPurify on PAAD (A) and THYM (B)

| 1. **PAAD** | XGBoost (all genes) | InfiniumPurify using DNA methylation data |
| --- | --- | --- |
| Predicted vs. ABSOLUTE estimated tumor purity values for the 53 PAAD test set samples | 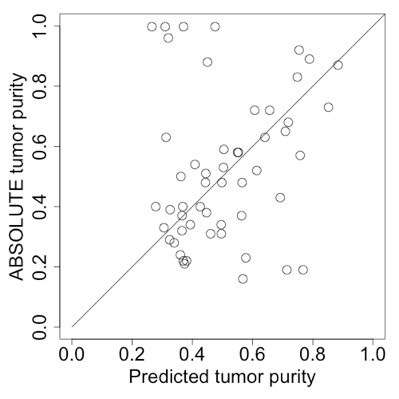 | 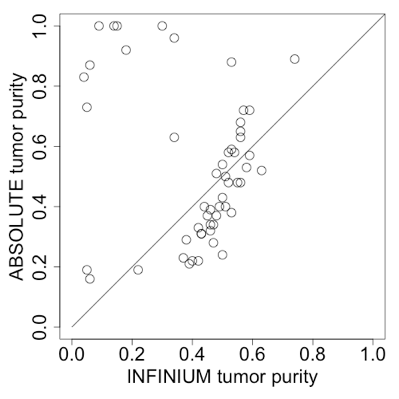 |
| RMSE | 0.27 | 0.34 |
| Pearson correlation | 0.21 | -0.19 |

| **(B) THYM** | XGBoost (all genes) | InfiniumPurify using DNA methylation data |
| --- | --- | --- |
| Predicted vs. ABSOLUTE estimated tumor purity values for the 27 THYM test set samples | 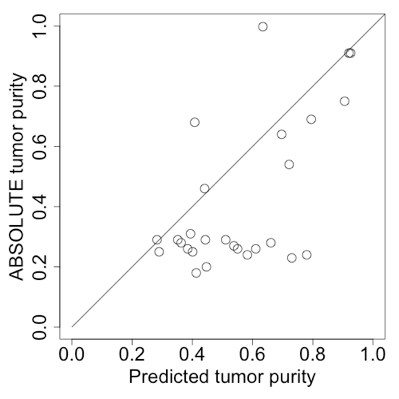 | 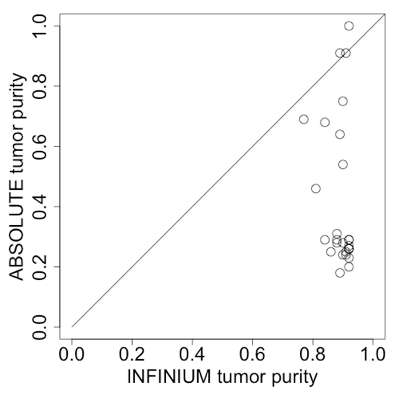 |
| RMSE | 0.24 | 0.53 |
| Pearson correlation | 0.62 | -0.21 |
