# Supplementary material for: Putative biomarkers for predicting tumor sample purity based on gene expression data
Source: BMC Genomics. 2019 Dec 27;20:1021. doi: 10.1186/s12864-019-6412-8 (PMC6933652; doi:10.1186/s12864-019-6412-8)
Supplement: Supplementary file 9 — Additional file 9: Table S6. Performance comparison between ESTIMATE and XGBoost for the test set samples [file 12864_2019_6412_MOESM9_ESM.docx]

**Table S6**. Performance comparison between ESTIMATE and XGBoost for the test set samples

| Method | ESTIMATE  (282 genes) | XGBoost  (all genes) | XGBoost  (ten marker genes) |
| --- | --- | --- | --- |
| Predicted vs. ABSOLUTE tumor purity values for the 2,359 test set samples | 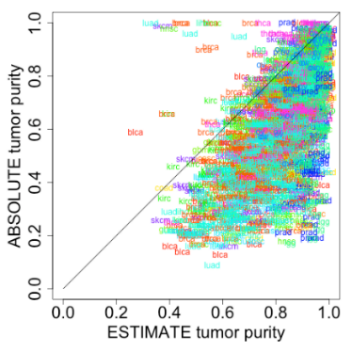 | 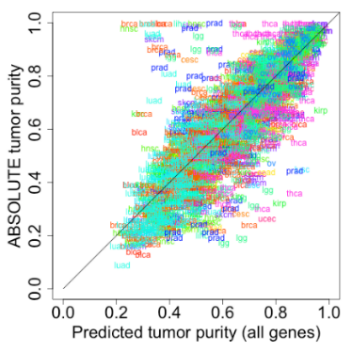 | 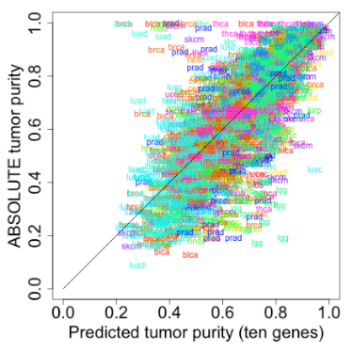 |
| RMSE | 0.25 | 0.12 | 0.14 |
| Pearson | 0.61 | 0.82 | 0.73 |
| Spearman | 0.63 | 0.83 | 0.74 |
