# Supplementary material for: Putative biomarkers for predicting tumor sample purity based on gene expression data
Source: BMC Genomics. 2019 Dec 27;20:1021. doi: 10.1186/s12864-019-6412-8 (PMC6933652; doi:10.1186/s12864-019-6412-8)
Supplement: Supplementary file 10 — Additional file 10: Table S7. Hyper-parameter search for Random Forrest models. [file 12864_2019_6412_MOESM10_ESM.docx]

**Table S7**. Hyper-parameter search for Random Forrest models.

| Parameter name | Parameter value considered | Parameter value selected |
| --- | --- | --- |
| Number of trees | {500, 1000} | 1000 |
| Fraction of feature used (per split) | [0.4, 1.0] | 1.0 |
| Fraction of sample used (per bag) | [0.4, 1.0] | 0.6 |
| Minimum leaf size | 5 | 5 |
