# Supplementary material for: Putative biomarkers for predicting tumor sample purity based on gene expression data
Source: BMC Genomics. 2019 Dec 27;20:1021. doi: 10.1186/s12864-019-6412-8 (PMC6933652; doi:10.1186/s12864-019-6412-8)
Supplement: Supplementary file 11 — Additional file 11: Table S8. Summary of TNBC sample purity prediction performance of individual XGBoost models across 1000 training-validation partitions. (A) using all genes; (B) using only the ten marker genes. [file 12864_2019_6412_MOESM11_ESM.docx]

**Table S8**. Summary of TNBC sample purity prediction performance of individual XGBoost models across 1,000 training-validation partitions. (A) using all genes; (B) using only the ten marker genes

| Procedure | Mean (S.D.) | | Median | |
| --- | --- | --- | --- | --- |
|  | RMSE | Pearson correlation | RMSE | Pearson correlation |
| 1. Using all genes as predictors | | | | |
| Training | 0.023 (0.006) | 0.994 (0.003) | 0.022 | 0.995 |
| Cross-validation | 0.136 (0.034) | 0.704 (0.184) | 0.133 | 0.749 |
| Testing | 0.135 (0.018) | 0.970 (0.014) | 0.134 | 0.972 |
|  |  |  |  |  |
| 1. Using only the ten marker genes as predictors | | | | |
| Training | 0.099 (0.004) | 0.858 (0.011) | 0.100 | 0.857 |
| Cross-validation | 0.137 (0.037) | 0.702 (0.192) | 0.133 | 0.749 |
| Testing | 0.239 (0.014) | 0.875 (0.03) | 0.240 | 0.877 |
