# Supplementary material for: Putative biomarkers for predicting tumor sample purity based on gene expression data
Source: BMC Genomics. 2019 Dec 27;20:1021. doi: 10.1186/s12864-019-6412-8 (PMC6933652; doi:10.1186/s12864-019-6412-8)
Supplement: Supplementary file 12 — Additional file 12: Figure S3. Scatter plot of XGBoost predicted and ABSOLUTE estimated tumor purity values for the 162 test set microarray samples. [file 12864_2019_6412_MOESM12_ESM.docx]

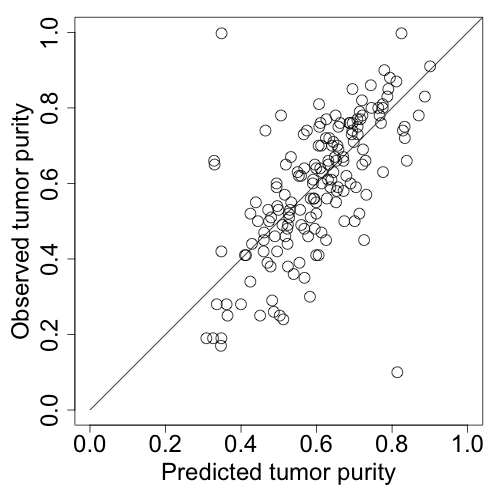


**Figure S3**. Scatter plot of XGBoost predicted and ABSOLUTE estimated tumor purity values for the 162 test set microarray samples.
