# Supplementary material for: Putative biomarkers for predicting tumor sample purity based on gene expression data
Source: BMC Genomics. 2019 Dec 27;20:1021. doi: 10.1186/s12864-019-6412-8 (PMC6933652; doi:10.1186/s12864-019-6412-8)
Supplement: Supplementary file 13 — Additional file 13: Figure S4. A schematic of our performance evaluation strategy. [file 12864_2019_6412_MOESM13_ESM.docx]

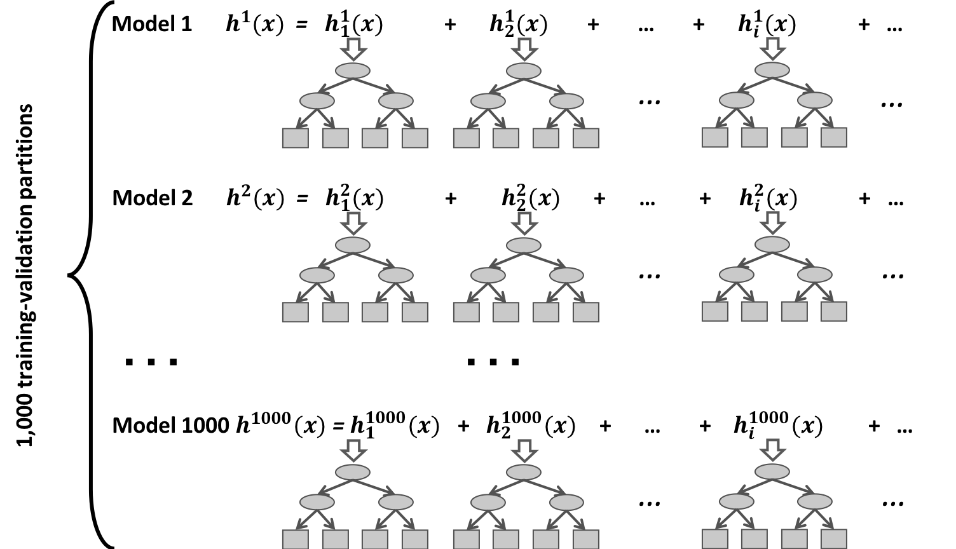


**Figure S4**. A schematic diagram of our performance evaluation strategy. Each row depicts a model fitted to the training set of a training-validation partition. The 1,000 training-validation partitions correspond to 100 repetitions of 10-fold cross-validation where 10% of the samples were sequentially set aside as testing samples and the remaining 90% of the samples as training samples. Each repetition was created stochastically by randomly selecting the samples without replacement. We used XGBoost to fit each model, a sequence of boosted trees. In a tree, ovals represent nodes and rectangles represent leaves. Tree *i* in the sequence for model *m* $h_{i}^{m}(x)$ , is a function of input data $x$, namely, gene expression levels. The first tree finds the best linear regression model that predicts a value of tumor purity given known values of the gene expression for all genes. Each subsequent tree fits the residuals of the tumor purity values that could not be explained by the tree before it.
