# Supplementary material for: Putative biomarkers for predicting tumor sample purity based on gene expression data
Source: BMC Genomics. 2019 Dec 27;20:1021. doi: 10.1186/s12864-019-6412-8 (PMC6933652; doi:10.1186/s12864-019-6412-8)
Supplement: Supplementary file 14 — Additional file 14: Figure S5. Box plots of RMSE of the predicted versus the observed tumor purity values for various number of models for the pan-cancer data. [file 12864_2019_6412_MOESM14_ESM.docx]

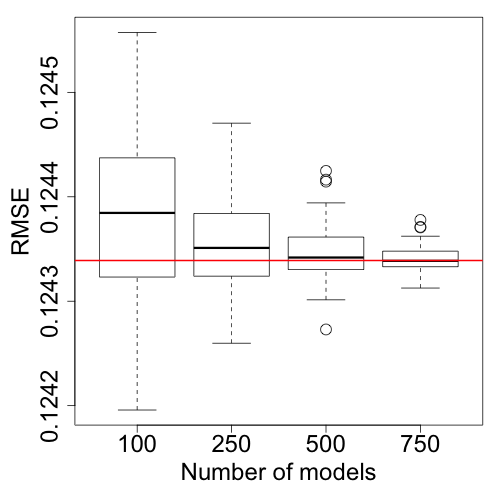


**Figure S5**. Box plots of RMSE of the predicted vs the observed tumor purity values for various number of models for the pan-cancer data.
