# Supplementary material for: Putative biomarkers for predicting tumor sample purity based on gene expression data
Source: BMC Genomics. 2019 Dec 27;20:1021. doi: 10.1186/s12864-019-6412-8 (PMC6933652; doi:10.1186/s12864-019-6412-8)
Supplement: Supplementary file 15 — Additional file 15: Table S9. XGBoost tuning parameters and those selected as the optimal set. [file 12864_2019_6412_MOESM15_ESM.docx]

**Table S9**. XGBoost tuning parameters and those selected as the optimal set

| Parameter | Considered | Selected |
| --- | --- | --- |
| Maximal number of trees | 5,000 | 5,000  (subject to early stopping criterion) |
| Maximal number of trees with no improvement (early stopping criterion) | 5 | 5 |
| Learning rate | [0.001, 0.1] | 0.05 |
| Maximum tree depth | [4, 10] | 4 |
| Minimum leaf weight | [1, 15] | 1 |
| Minimum split gain | 0 | 0 |
| Fraction of features used | [0.5, 1.0] | 0.65 |
| Fraction of samples used | [0.4, 1.0] | 0.85 |
